# How accurate is 18F-FDG-PET-CT in determining local cartilaginous/bony involvement by head & neck malignancy?

**DOI:** 10.1186/1470-7330-14-S1-P1

**Published:** 2014-10-09

**Authors:** LI Sonoda, A Lakhani, S Ghosh-Ray

**Affiliations:** 1 Paul Strickland Scanner Centre, Mount Vernon Hospital, London, UK

## Aims

18F-FDG-PET-CT plays an important role in the management of head and neck cancers (HNC). In particular, presence/absence of local osseous invasion is an important factor in T-staging and determining treatment options. This study aimed to determine the accuracy of PET-CT in prediction of local osseous invasion by head and neck cancers.

## Methods

A 6-year-period retrospective analysis of 771 PET-CT scans of HNC (oral/nasal cavity, pharynx, larynx) was performed. Final diagnosis of osseous involvement was determined by histopathology, clinical and imaging follow-up.

## Results

PET-CT scans demonstrated increased abnormal osseous uptake in 63 cases, of which 52 were true osseous invasion, but 11 were false-positive (4 due to osteoradionecrosis, 4 benign dental infection/inflammation, 3 over-staging due to intense FDG-uptake nearby the bone). 708 cases were reported as ‘no osseous uptake’, of which 704 were true-negative, but 4 were false-negative (2 due to intrinsically low FDG-avid primary disease and bony lesions were not significantly FDG-avid, 2 due to bony necrosis of tumour with no significant FDG-uptake).

Sensitivity, specificity, PPV, NPV and accuracy of PET-CT in detecting local osseous invasion are 93, 98, 83, 99 and 98% respectively.

## Conclusion

18F-FDG-PET-CT plays an important role in detecting local osseous invasion by HNC, with an accuracy of 98%. Important false-positives are due to benign causes such as infection and osteoradionecrosis, and due to intense FDG-uptake nearby the bone. If there is clinical doubt further investigations including MRI and biopsy should be performed.

